# *MCM2* and *NUSAP1* Are Potential Biomarkers for the Diagnosis and Prognosis of Pancreatic Cancer

**DOI:** 10.1155/2020/8604340

**Published:** 2020-04-28

**Authors:** Yajun Deng, Hanyun Ma, Jinyong Hao, Qiqi Xie, Ruochen Zhao

**Affiliations:** ^1^Lanzhou University, Lanzhou, Gansu Province 730030, China; ^2^Department of Orthopaedics, Lanzhou University Second Hospital, Lanzhou, Gansu Province 730030, China; ^3^Comprehensive Cancer Center, Charité Universitätsmedizin Berlin, 10117 Berlin, Germany; ^4^Department of Gastroenterology, Lanzhou University Second Hospital, Lanzhou, Gansu Province 730030, China; ^5^Department of Critical Care Medicine, Lanzhou University Second Hospital, Lanzhou, Gansu Province 730030, China

## Abstract

Pancreatic cancer (PC) is one of the most malignant tumors. Despite considerable progress in the treatment of PC, the prognosis of patients with PC is poor. The aim of this study was to identify potential biomarkers for the diagnosis and prognosis of PC. First, the original data of three independent mRNA expression datasets were downloaded from the Gene Expression Omnibus and The Cancer Genome Atlas databases and screened for differentially expressed genes (DEGs) using the R software. Subsequently, Gene Ontology (GO) and Kyoto Encyclopedia of Genes and Genomes pathway enrichment analyses of the DEGs were performed, and a protein-protein interaction (PPI) network was constructed to screen for hub genes. The hub genes were analyzed for genetic variations, as well as for survival, prognostic, and diagnostic value, using the cBioPortal and Gene Expression Profiling Interactive Analysis (GEPIA) databases and the pROC package. After screening for potential biomarkers, the mRNA and protein levels of the biomarkers were verified at the tissue and cellular levels using the Cancer Cell Line Encyclopedia, GEPIA, and the Human Protein Atlas. As a result, a total of 248 DEGs were identified. The GO terms enriched in DEGs were related to the separation of mitotic sister chromatids and the binding of the spindle to the extracellular matrix. The enriched pathways were associated with focal adhesion, ECM-receptor interaction, and phosphatidylinositol 3-kinase (PI3K)/AKT signaling. The top 20 genes were selected from the PPI network as hub genes, and based on the analysis of multiple databases, MCM2 and NUSAP1 were identified as potential biomarkers for the diagnosis and prognosis of PC. In conclusion, our results show that MCM2 and NUSAP1 can be used as potential biomarkers for the diagnosis and prognosis of PC. The study also provides new insights into the underlying molecular mechanisms of PC.

## 1. Introduction

Pancreatic cancer (PC) is one of the most common malignant tumors, with a 5-year survival rate of only 9% [1]. Currently, surgery is the most effective way to improve the survival rate of patients with PC. However, the prognosis of patients with PC is still very poor because the onset of PC is cryptic, symptoms are atypical, lymph node metastasis occurs early, the degree of malignancy is high, and the progress is rapid [2]. Therefore, early diagnosis and intervention are essential for reducing mortality and improving the clinical prognosis of patients with PC.

The main potential biomarkers of PC identified in the past two decades are CA19-9, DUPAN-2, CAM17.1, TPS, SPan-1, TAT1, POA, YKL-40, TUM2-PK, and matrix metalloproteinases [3]. Although CA19-9, which is considered a better biomarker for the diagnosis and prognosis of PC [4], is highly sensitive, its application in early diagnostic screening for PC is limited owing to a low specificity [5, 6]. Therefore, research to find effective biomarkers for PC continues.

Increasing evidence indicates that abnormal expression of and mutations in certain genes are closely related to the occurrence and development of PC. Abnormal expression of *OPN* and *CISD2* has been shown to play a key role in the progression of PC [7, 8], while PAM4, S100A6, and SPARC have been identified as biomarkers of PC [9–11]. Therefore, further elucidation of the pathogenesis of PC at the genetic level may help identify new diagnostic and prognostic indicators. With the rapid development of sequencing technology, microarray analyses, based on high-throughput platforms, have been widely used in biomedical and clinical research for screening genetic variants [12, 13]. At present, there are many PC-related expression profile datasets of varying quality. However, most of the PC-related bioinformatics studies based on The Cancer Genome Atlas (TCGA) and Gene Expression Omnibus (GEO) did not perform quality control of the original data, nor did they verify their findings in other databases. Consequently, potential diagnostic and prognostic biomarkers showing compatibility across different transcriptomic platforms and patient cohorts have not been systematically investigated. In this study, we aimed to identify potential diagnostic and prognostic biomarkers for PC using the data available in the GEO and TCGA databases and validate the expression of these biomarkers using the Cancer Cell Line Encyclopedia (CCLE), International Cancer Genome Consortium (ICGC), cBioPortal, Gene Expression Profiling Interactive Analysis (GEPIA), and Human Protein Atlas databases. Our results will help develop novel therapeutic strategies to improve clinical outcomes and provide new insights into the pathogenesis of PC.

## 2. Materials and Methods

### 2.1. Gene Expression Profile Datasets

GEO (http://www.ncbi.nlm.nih.gov/geo) is a public repository of various high-throughput experimental data [14]. In this study, two mRNA expression profile datasets for PC (GSE15471 [15] and GSE16515 [16]), based on the GPL570 platform (Affymetrix Human Genome U133 Plus 2.0 Array), were downloaded from the GEO database. The GSE15471 dataset consisted of 39 PC tissues and 21 normal pancreatic tissues, and the GSE16515 dataset included 36 PC tissues and 16 normal pancreatic tissues. TCGA (http://www.cancergenome.nih.gov) is a large-scale cancer genetic information database [17] that provides information regarding the key genomic changes and clinical data for 33 cancers. An mRNA expression profile that contained data for 179 PC tissues and four normal pancreatic tissues was obtained from TCGA.

### 2.2. Data Preprocessing and Identification of Differentially Expressed Genes (DEGs)

The affy package [18] in the R software (version 3.6.1, http://r-project.org/) was used to read the raw data (CEL file) of the three datasets and then convert the original data format, fill the missing values, and apply background correction. The samples were then subjected to differential expression analysis, and the DEGs were exported using the limma package [19]. DEGs that satisfied the adj. *P* value < 0.05 and ∣log_2_FC∣ > 1 criteria were considered. Finally, the intersection of the DEGs from the three datasets was obtained using the FunRich software [20] (version 3.1.3, http://funrich.org/index.html).

### 2.3. Construction of the Protein-Protein Interaction (PPI) Network and Analysis of Important Modules

The Search Tool for the Retrieval of Interacting Genes database (version 11.0, http://string-db.org) [21] was used to construct a PPI network for DEGs, and interactions with a combined score of greater than 0.9 were considered statistically significant. The PPI network was then visualized using the Cytoscape software (version 3.7.1) [22]. To better extract valuable clues from important modules, the cytoHubba plugin [23] was used to select and sort 20 genes using the Maximum Correlation Criteria (MCC) algorithm. The NetworkAnalyst database (https://www.networkanalyst.ca/faces/home.xhtml) was used to display the coexpressed gene network of DEGs; cBioPortal (https://www.cbioportal.org) [24] was used to analyze the hub genes and their coexpressed genes in PC; the Cancer RNA-Seq Nexus (CRN) database (http://syslab4.nchu.edu.tw) [25] was used to further validate the expression of the hub genes.

### 2.4. Gene Ontology (GO) and Kyoto Encyclopedia of Genes and Genomes (KEGG) Pathway Enrichment Analyses of DEGs

GO covers three aspects of biology: cellular component (CC), molecular function (MF), and biological process (BP) [26]. KEGG is a database that analyzes high-level functions of biological systems at the molecular level [27]. To further analyze the functions of DEGs, GO and KEGG pathway enrichment analyses of DEGs were performed using the clusterProfiler package [28]. FDR < 0.05 was considered statistically significant.

### 2.5. GO and KEGG Pathway Enrichment Analyses of the Hub Genes

The Database for Annotation, Visualization, and Integrated Discovery (DAVID; https://david.ncifcrf.gov; version 6.8) provides a comprehensive set of gene and protein functional annotation information [29]. GO and KEGG pathway enrichment analyses of the hub genes were performed using the DAVID database, and FDR < 0.05 was considered statistically significant.

### 2.6. Screening for Biomarkers

To further screen for potential biomarkers for the diagnosis and prognosis of PC, a comprehensive analysis of the 20 hub genes was performed. We used cBioPortal to analyze genetic variations in the hub genes. The GEPIA database (http://gepia.cancer-pku.cn) [30] was used to analyze associations of hub gene expression with overall survival (OS) and disease-free survival (DFS) of patients with PC. To further investigate the diagnostic value of the hub genes for PC, receptor operating characteristic (ROC) curves were plotted using the pROC package [31]. Potential biomarkers for the diagnosis and prognosis of PC were investigated by analyzing genetic changes, verifying differential expression, and evaluating the survival, prognostic, and diagnostic value of the hub genes.

### 2.7. Multidimensional Verification of Biomarkers

To minimize the bias and improve the accuracy of the results of analysis, multiple online databases, including CCLE (https://portals.broadinstitute.org/ccle) [32], the Human Protein Atlas (https://www.Proteinatlas.org/) [33], and GEPIA, were used to determine the mRNA and protein expression levels of potential biomarkers at the tissue and cell levels. The therapeutic potential of the biomarkers was investigated by analyzing genetic correlations between the screened biomarkers and *EGFR*, *ERBB2*, and *KRAS*, which are important therapeutic targets in PC [34–36], using the GEPIA database and circlize package in R [37].

## 3. Results

### 3.1. DEGs Identified in the Three Datasets

**Figure 1:**
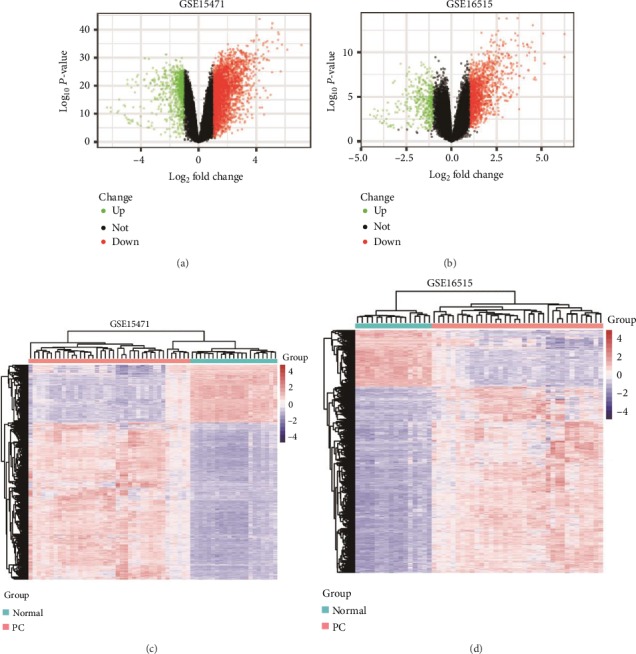
Differential expression analysis. (a) Volcano plot of DEGs in the GSE15471 dataset. (b) Volcano plot of DEGs in the GSE16515 dataset. Red dots indicate upregulated genes; green dots indicate downregulated genes; and black dots indicate unaltered genes. (c) Hierarchical clustering heatmap of DEGs in the GSE15471 dataset. (d) Hierarchical clustering heatmap of DEGs in the GSE16515 dataset. Blue represents downregulated genes, and red represents upregulated genes.

**Figure 2:**
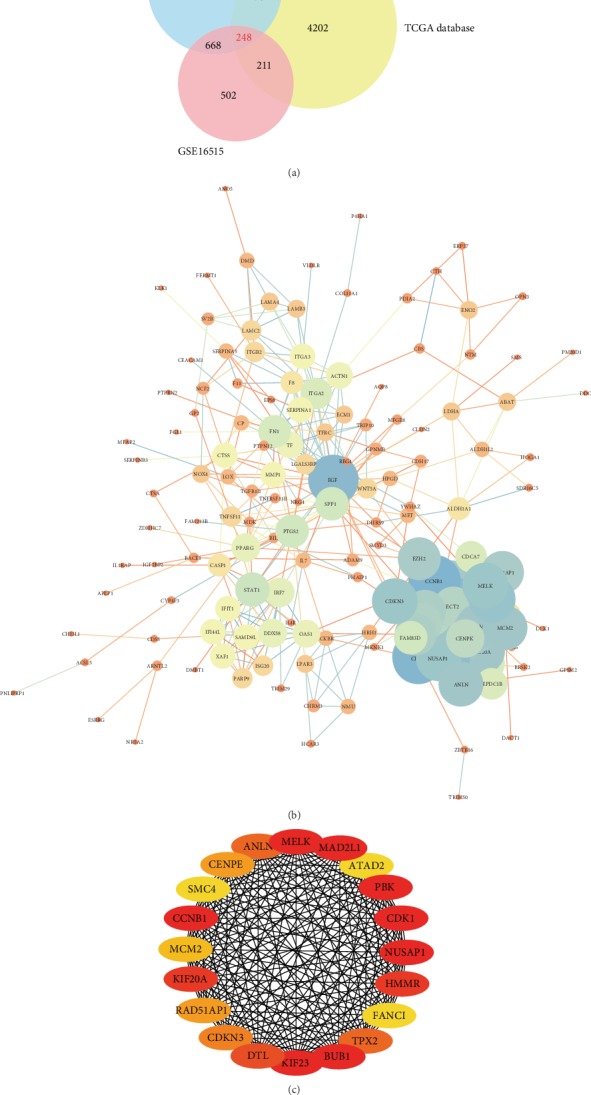
Venn diagram and the PPI network. (a) Venn diagram of the overlapping DEGs in the three datasets. (b) PPI network of DEGs. The size of the circle indicates the weight of the gene action; the line indicates the interaction between the genes, and the thickness of the line indicates the strength of the interaction. (c) Twenty hub genes. A darker color represents a higher score, and a lighter color represents a lower score.

The results of normalization of the sample data from the GSE15471 and GSE16515 datasets are presented in box plots (Fig. S1a and S1b). Samples in both datasets were at the same level, indicating high consistency. The results of the sample cluster analysis of the two datasets are shown in Fig. S1c and S1d, indicating that the sample quality was reliable. After data preprocessing, we extracted 2,759 and 1,629 DEGs from the GSE15471 and GSE16515 mRNA expression profiles, respectively, using the R software. The volcano plots of the upregulated and downregulated DEGs are shown in Figures 1(a) and 1(b). Figures 1(c) and 1(d) show the hierarchical clustering heatmaps of DEGs from the two mRNA expression profiles. A total of 5,134 DEGs were obtained from the mRNA expression profiles derived from TCGA database. The DEGs from the three datasets were then intersected, and 248 DEGs were found to overlap among the three datasets (Figure 2(a)).

### 3.2. Analysis of Important Modules in the PPI Network

**Figure 3:**
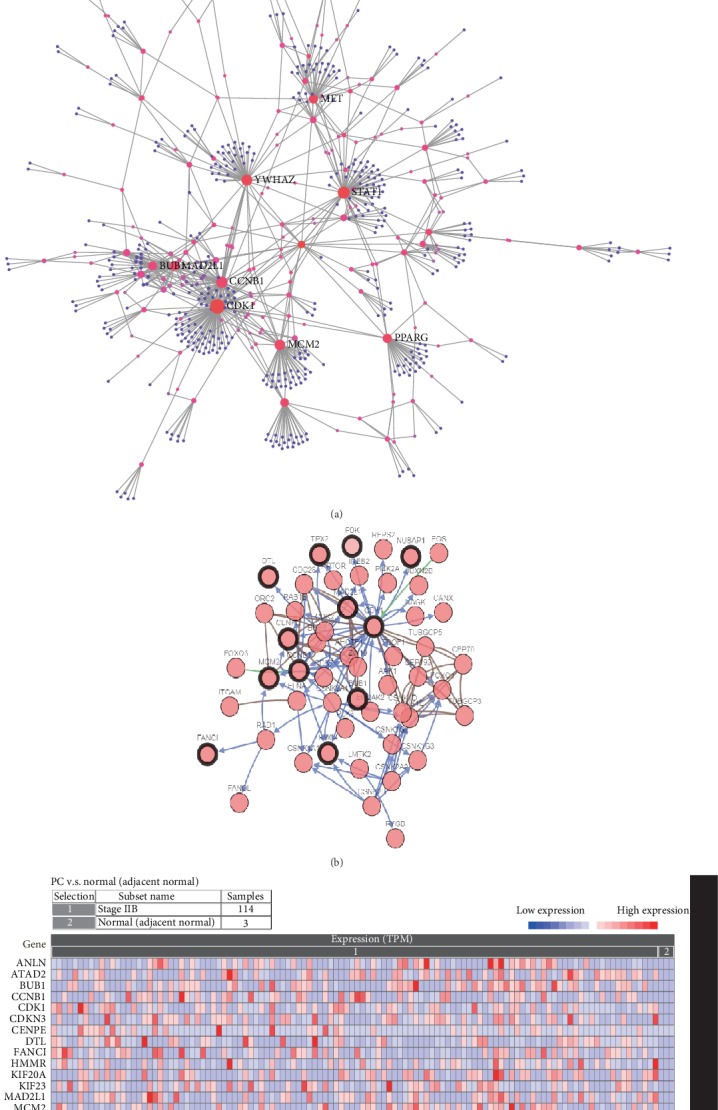
DEGs and hub gene coexpression networks. (a) Coexpression gene networks of DEGs. (b) Network of interactions between the hub genes and their coexpressed genes in PC. (c) Heatmap verifying the expression of the hub genes in the CRN database. Red indicates high expression of the gene, and blue indicates low expression of the gene.

The PPI network for the DEGs is presented in Figure 2(b). The top 20 genes, which were selected as the hub genes using the most relevant standard (MCC), included *MELK*, *MAD2L1*, *ATAD2*, *PBK*, *CDK1*, *NUSAP1*, *HMMR*, *FANCI*, *TPX2*, *BUB1*, *KIF23*, *DTL*, *CDKN3*, *RAD51AP1*, *KIF20A*, *MCM2*, *CCNB1*, *SMC4*, *CENPE*, and *ANLN* (Figure 2(c) and Table 1). The coexpression gene network of the DEGs is presented in Figure 3(a). The DEGs had a large weight ratio in the coexpression network, with a large number of related genes, indicating that the DEGs play a comprehensive and complex role in the pathogenesis of PC. Simultaneously, the hub genes and their coexpression gene network in PC (Figure 3(b)) were analyzed using cBioPortal. The results showed that the 20 hub genes were closely related to and interacted with their coexpressed genes. The heatmap of the 20 hub genes, constructed using the CRN database, is shown in Figure 3(c). These 20 hub genes were highly expressed in PC tissues but not in normal tissues.

### 3.3. GO and KEGG Pathways Enriched in DEGs

**Figure 4:**
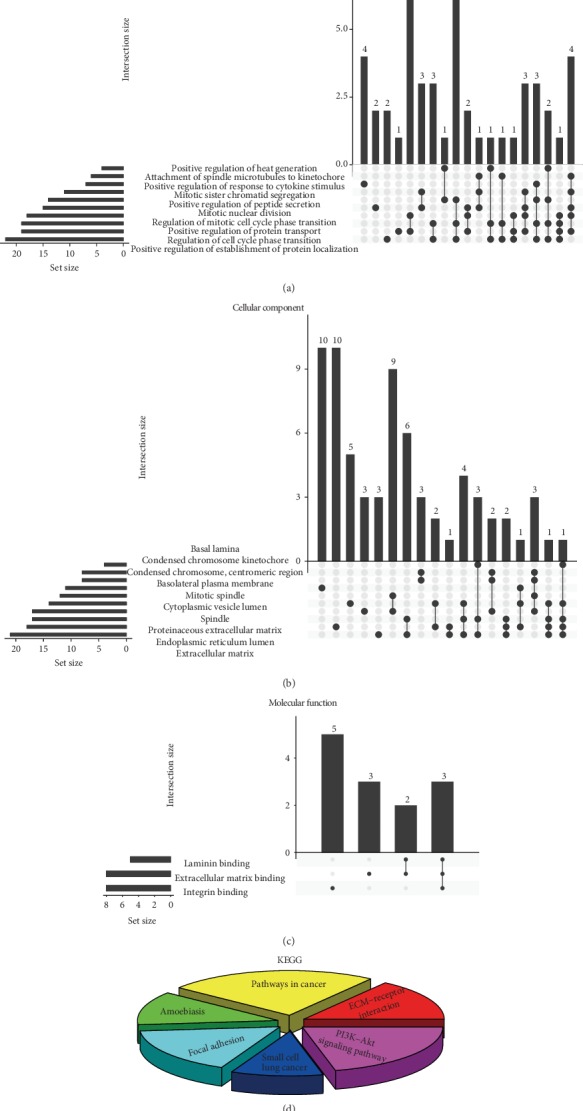
GO and KEGG pathway enrichment analyses of DEGs. (a) BP, (b) CC, (c) MF, and (d) KEGG analysis results.

GO analysis showed that BP-related changes in DEGs were significantly enriched in mitotic sister chromatid separation, regulation of cell cycle phase transition, and positive regulation response to cytokine stimulation (Figure 4(a)). The changes in CC were mainly in the centromere region, spindle, and endoplasmic reticulum cavity (Figure 4(b)). The changes in MF were mainly enriched in the extracellular matrix binding and integrin binding (Figure 4(c)). The KEGG pathway enrichment analysis showed that the enriched pathways were associated with focal adhesion, ECM-receptor interaction, and the phosphatidylinositol 3-kinase (PI3K)/Akt signaling pathway (Figure 4(d)).

### 3.4. GO and KEGG Pathways Enriched in the Hub Genes

The GO terms enriched in the hub genes were mainly related to the apoptosis process, cell cycle, chromosome, and histone kinase activity, while the KEGG pathways enriched in the hub genes were related to the cell cycle and p53 signaling pathway (Fig. S2).

### 3.5. Identification of *MCM2* and *NUSAP1* as Potential Biomarkers

**Figure 5:**
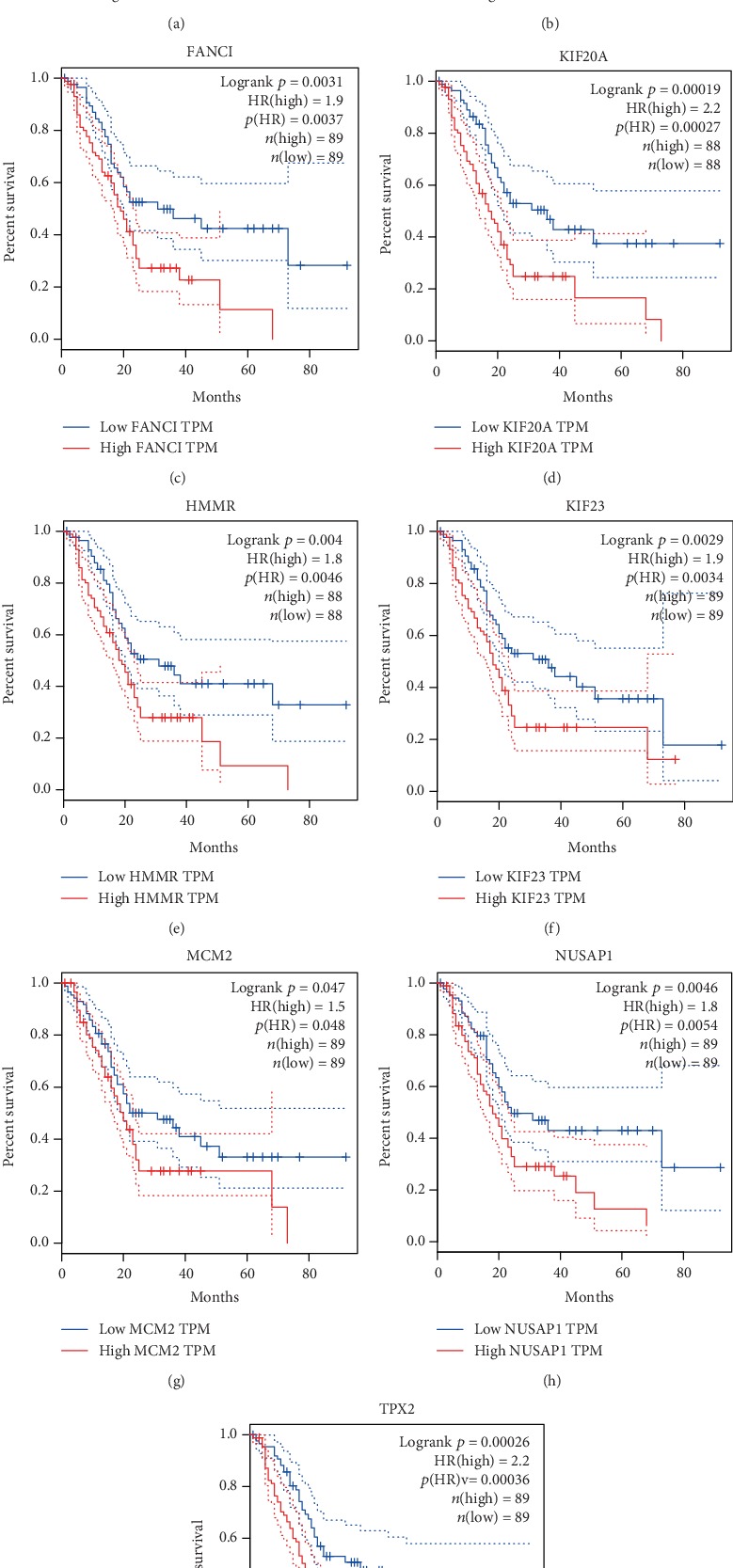
Association of hub gene expression with overall survival of patients. (a) *BUB1*, (b) *CDK1*, (c) *FANCI*, (d) *KIF20A*, (e) *HMMR*, (f) *KIF23*, (g) *MCM2*, (h) *NUSAP1*, and (i) *TPX2*. The solid line represents the survival curve, and the dashed line represents the 95% confidence interval. Patients with higher than the median value are indicated by the red line, and those with lower than the median value are indicated by the blue line. Log-rank *P* < 0.05 was considered statistically significant.

**Figure 6:**
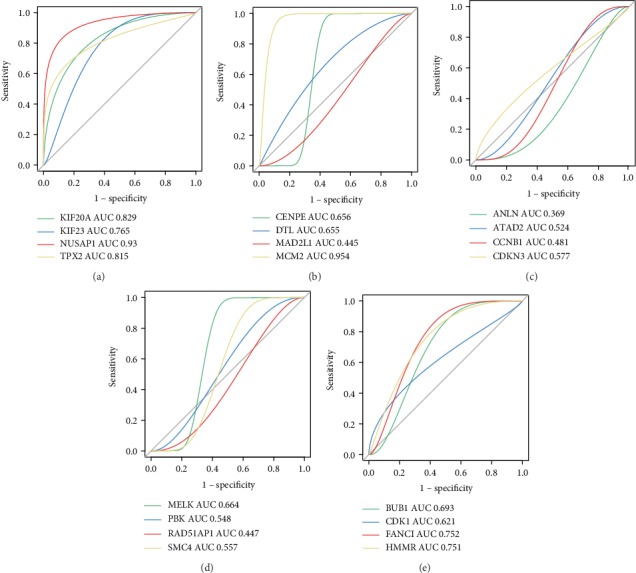
Analysis of the diagnostic value of the hub genes using ROC curve analysis. (a) *KIF20A*, *KIF23*, *NUSAP1*, and *TPX2*. (b) *CENPE*, *DTL*, *MAD2L1*, and *MCM2*. (c) *ANLN*, *ATAD2*, *CCNB1*, and *CDKN3*. (d) *MELK*, *PBK*, *RAD51AP1*, and *SMC4*. (e) *BUB1*, *CDK1*, *FANCI*, and *HMMR*. AUC represents the area under the ROC curve.

To screen for potential biomarkers for the diagnosis and prognosis of PC, we first used cBioPortal to analyze whether genetic variations in the hub genes are involved in PC progression. As shown in Fig. S3, the 20 hub genes showed genetic variations in the PC samples. Among the genes, the highest genetic variation rate was found in *ATAD2* (5%), including missense, start lost, initiator codon, frameshift, stop lost, and stop gained mutations, which suggested that these mutations might be involved in the occurrence and progression of PC. To investigate the prognostic value of the hub genes in PC, we performed a survival analysis using the GEPIA database. As shown in Figure 5, high mRNA levels of *BUB1*, *CDK1*, *FANCI*, *KIF20A*, *HMMR*, *KIF23*, *MCM2*, *NUSAP1*, and *TPX2* were associated with a poor OS and were also closely related to a poor DFS of patients (Fig. S4). Thus, the results of the survival analysis suggested that these nine hub genes might be new indicators for predicting the prognosis of PC. The results obtained using ROC curve analysis showed that *MCM2* (area under the curve (AUC) = 0.954) and *NUSAP1* (AUC = 0.93) had the highest diagnostic value for PC (Figure 6). In summary, we identified *MCM2* and *NUSAP1* as potential biomarkers for the diagnosis and prognosis of PC using analysis of genetic variation, verification of differential expression, and evaluation of the survival, prognostic, and diagnostic value of the 20 hub genes.

### 3.6. Multidimensional Verification of MCM2 and NUSAP1 as Potential Biomarkers

**Figure 7:**
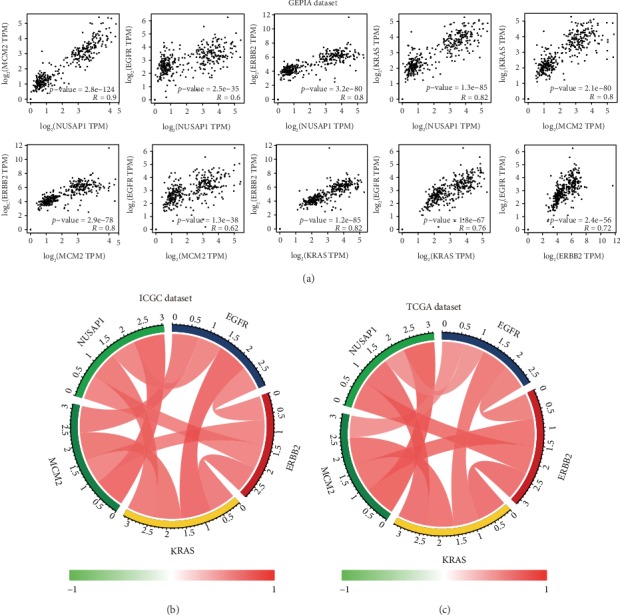
Intergene correlation analysis of expression levels based on (a) the GEPIA database, (b) the ICGC database, and (c) TCGA database.

**Figure 8:**
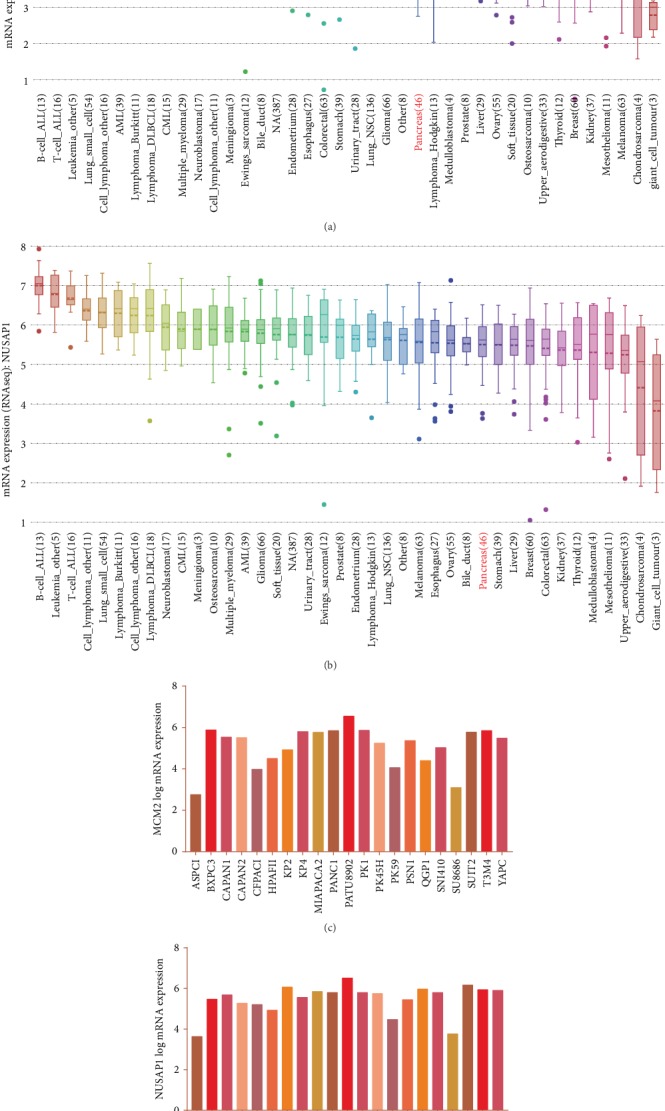
Expression of *MCM2* and *NUSAP1* at the cell level. (a) Expression of *MCM2* in cell lines derived from different tissues. (b) Expression of *NUSAP1* in cell lines derived from different tissues. (c) Expression of *MCM2* in 21 commonly used PC cell lines. (d) Expression of *NUSAP1* in 21 commonly used PC cell lines.

**Figure 9:**
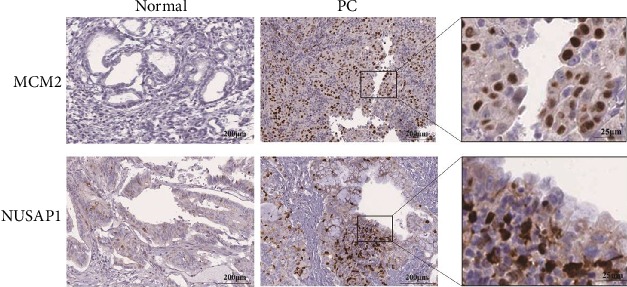
Representative immunohistochemical images of MCM2 and NUSAP1 expression in normal pancreatic and PC tissues.

To ensure the accuracy of the analytical results, we used multiple databases to validate the mRNA and protein expression of MCM2 and NUSAP1 in PC at the tissue and cell levels. We used the GEPIA (Figure 7(a)), ICGC (Figure 7(b)), and TCGA (Figure 7(c)) databases to analyze the *MCM2*, *NUSAP1*, *EGFR*, *ERBB2*, and *KRAS* expression. The correlation coefficient (*R*) values are shown in Table 2. At the cell level, CCLE was used to analyze the expression of *MCM2* and *NUSAP1* in cell lines derived from different tissues. The results showed that *MCM2* and *NUSAP1* were expressed in various cell lines, but their mRNA levels were low in pancreatic cell lines (Figures 8(a) and 8(b)). We also analyzed the mRNA expression of *MCM2* and *NUSAP1* in 21 experimental PC cell lines and found that both were upregulated in most of the PC cell lines (Figures 8(c) and 8(d)). At the tissue level, the GEPIA-based analysis indicated that MCM2 and NUSAP1 were upregulated in PC tissues (Fig. S5). Further analysis using the Human Protein Atlas showed that MCM2 and NUSAP1 were poorly expressed at the mRNA and protein levels in normal pancreatic tissues compared with their expression in other tissues (Fig. S6). The MCM2 and NUSAP1 protein levels were higher in PC tissues than in normal pancreatic tissues (Figure 9).

## 4. Discussion

The lack of methods for early screening for and detection of PC leads to late detection of the disease and a high mortality rate in patients [38]. Hence, early diagnosis and treatment are pivotal for improving the clinical outcome of patients with PC. A growing body of evidence suggests that some of dysregulated genes in PC may be potential biomarkers for the diagnosis and prognosis of the disease [39, 40]. Therefore, we used bioinformatics tools to analyze PC-associated mRNA expression profiles and to identify potential biomarkers for diagnosis and prognosis of PC.

In this study, we downloaded three PC-related mRNA expression datasets from the GEO and TCGA databases and screened the data for DEGs. In total, 248 DEGs were identified, and it was found that the GO terms enriched in DEGs were mainly related to “mitotic mitosis”, “centromere region”, “mitotic spindle”, and “extracellular matrix binding”; the enriched pathways were mainly associated with “focus adhesion”, “ECM-receptor interactions”, and “PI3K/Akt signaling pathway”. ECM and focal adhesion have been shown to be important components of tumorigenesis and cancer progression [41–43]. Dysregulation of the cell cycle is the key factor in the malignant biological behaviors associated with the proliferation, invasion, and metastasis of PC cells [44, 45]. Zhang et al. [46] showed that LAMB3 affected the proliferation, invasion, and metastasis of PC by regulating the PI3K/Akt signaling pathway. Our data are consistent with the above findings and provide new insights into molecular mechanisms of pathogenesis of PC.

In addition, we constructed a PPI network for DEGs and selected the top 20 genes as the hub genes. The hub genes were analyzed using GO and KEGG pathway enrichment and were found to be mainly enriched in the GO terms “apoptotic process” and “cell cycle” and in the KEGG pathways related to the “cell cycle” and “p53 signaling pathway”. Previous studies have reported that the apoptotic process and cell cycle are closely related to the development and progression of PC [47, 48]. As one of the important signaling pathways in the body, the p53 signaling pathway has been shown to be involved in the development, invasion, and metastasis of various tumors [49, 50]. Studies have reported that targeting of DTL can induce cell cycle arrest and senescence and can therefore be used to treat liver cancer [51]. Downregulation of MELK [52], MAD2L1 [53], and CCNB1 [54] can also inhibit cell cycle progression of liver cancer. ANLN [55], BUB1 [56], CDK1 [57], SMC4 [58], CENPE [59], ATAD2 [60], TPX2 [61], KIF23 [62], CDKN3 [63], and KIF20A [64] are closely related to the development of various tumors. However, reports regarding the role of *FANCI* and *RAD51AP1* in tumors are limited, and hence, these genes deserve further investigation.

To further screen for potential biomarkers for the diagnosis and prognosis of PC, we analyzed genetic variations, verified the differential expression, and evaluated the survival, prognostic, and diagnostic value of the hub genes. Finally, we identified *MCM2* and *NUSAP1* as potential biomarkers for the diagnosis and prognosis of PC. To minimize the bias, we used multiple databases to verify the mRNA and protein levels of MCM2 and NUSAP1 at the tissue and cell levels. The results showed that the mRNA and protein levels of MCM2 and NUSAP1 were significantly upregulated in PC cells and tissues. In addition, gene correlation analysis showed that the expression levels of *MCM2*, *NUSAP1*, *EGFR*, *ERBB2*, and *KRAS* significantly correlated, and thus, MCM2 and NUSAP1 are likely to become potential therapeutic targets for PC.

MCM2 is an important DNA replication initiation factor in humans. It is present in the nucleus and is highly expressed in proliferating cells. MCM2 is not expressed or is poorly expressed in quiescent or well-differentiated cells, suggesting that it can be used as a specific marker for proliferating cells. Studies have reported that MCM2 is a key molecule involved in the pathogenesis of non-small-cell lung cancer and may be a novel therapeutic target for lovastatin in the treatment of this cancer [65]. Proteomic analysis of clinical specimens of high-grade small cell lung cancer showed that MCM2 was associated with a poor prognosis in patients [66]. Increasing evidence has confirmed that MCM2 can be used as a biomarker for the diagnosis and prognosis of various cancers. Yang et al. [67] showed that MCM2 expression in gastric cancer tissues was significantly higher than that in the normal gastric mucosa, and the levels of expression positively correlated with the prognosis, suggesting that MCM2 can be used as a novel prognostic biomarker for gastric cancer. Torres-Rendon et al. [68] analyzed the MCM2 expression in clinical samples of oral squamous cell carcinoma using immunohistochemistry and demonstrated that MCM2 might be a useful prognostic marker for the disease. In another study, staining for MCM2 was directly performed on 183 liquid-based cytological samples. Studies have shown that the positive rate of MCM2 expression increased with the severity of cervical lesions and was related to the type of human papilloma virus, which indicates a potential application value of MCM2 in the diagnosis of cervical lesions [69]. All these results show that MCM2 is involved in the occurrence and progression of various tumors and can be a potential biomarker for their diagnosis and prognosis, which is consistent with the results of our analysis. However, there are no studies regarding the role of MCM2 in PC, and thus, MCM2 needs to be confirmed as a potential marker for the diagnosis and prognosis of PC.

NUSAP1 is a tubulin that is involved in the assembly of the spindle to ensure a normal cell cycle, thus playing an important role in mitosis [70]. An abnormal cell cycle is an important feature of tumor formation, and currently, the role of NUSAP1 in cancer is being actively investigated. Some studies have shown overexpression of NUSAP1 in renal cell carcinoma [71], colon cancer [72], glioma [73], and other malignant tumors and its significant association with tumor invasion and metastasis, as well as with a poor prognosis in patients. Thus, the results of this study are consistent with those regarding the role of NUSAP1 in other tumors. NUSAP1 expression in acute myeloid leukemia can block the cell cycle [74], whereas reduction in the NUSAP1 expression increased the killing effect of paclitaxel on oral epithelial squamous cell carcinoma cells [75]. In patients with liver cancer, the level of NUSAP1 expression is closely associated with the severity of the prognosis, whereas interference with NUSAP1 expression inhibits the growth of liver cancer cells [76]. Gulzar et al. [77] found that high levels of NUSAP1 expression were related to the growth characteristics of tumor cells in PC, thus making NUSAP1 a novel biomarker for PC recurrence after surgery. Studies have also confirmed that NUSAP1 has a high prognostic value for breast cancer [78]. Thus, the high prognostic value of NUSAP1 for various tumors is consistent with the results of this study. Although relevant research on the role of NUSAP1 in PC is lacking, we speculated that NUSAP1 may be a potential biomarker for PC.

Most bioinformatics studies have reported analysis of a single mRNA expression dataset. In this study, we selected three mRNA expression datasets from two databases, thereby increasing the sample size and confidence level. We used different bioinformatics methods to mine the data deeper and used multiple databases to perform multidimensional verification of MCM2 and NUSAP1 expression in PC, providing diverse perspectives. However, this study has certain limitations. First, a certain degree of heterogeneity was present in the datasets selected for this study, and only four normal samples were included in TCGA. Although we removed the batch data and performed quality control and standardization of the raw data, a larger sample size and a higher-quality dataset are still required to verify the reliability of the results. Second, our study involved a second round of mining and analysis of previously published datasets. Although some previous data were consistent with those of our analysis, further molecular biology experiments are required to verify the accuracy of our results. As a future research direction, validation of MCM2 and NUSAP1 as diagnostic and prognostic markers of PC is needed in a large number of clinical PC samples and PC cell lines.

## 5. Conclusions

In conclusion, using bioinformatics analysis of three mRNA expression datasets, a total of 20 hub genes were identified, which may play key roles in the occurrence and progression of PC. Analysis of the clinical significance of the 20 hub genes in PC revealed two abnormally regulated genes, *MCM2* and *NUSAP1*, which were confirmed, using multidimensional verification, as potential biomarkers for the diagnosis and prognosis of PC. However, the results need to be validated in a larger number of clinical samples using additional experimental methods before using MCM2 and NUSAP1 as effective diagnostic and prognostic markers for PC.

## Figures and Tables

**Table 1:** Top 20 hub genes ranked using the Maximum Correlation Criteria algorithm.

Rank	Gene symbol	Full name	Entrez ID	Score
1	*BUB1*	BUB1 mitotic checkpoint serine/threonine kinase	699	2.71*E* + 22
1	*CCNB1*	Cyclin B1	891	2.71*E* + 22
1	*CDK1*	Cyclin-dependent kinase 1	983	2.71*E* + 22
1	*KIF23*	Kinesin family member 23	9493	2.71*E* + 22
1	*MAD2L1*	Mitotic arrest deficient 2-like 1	4085	2.71*E* + 22
1	*MELK*	Maternal embryonic leucine zipper kinase	9833	2.71*E* + 22
1	*NUSAP1*	Nucleolar and spindle-associated protein 1	51203	2.71*E* + 22
1	*PBK*	PDZ-binding kinase	55872	2.71*E* + 22
9	*HMMR*	Hyaluronan-mediated motility receptor	3161	2.71*E* + 22
9	*KIF20A*	Kinesin family member 20A	10112	2.71*E* + 22
11	*DTL*	Denticleless E3 ubiquitin protein ligase homolog	51514	2.71*E* + 22
12	*ANLN*	Anillin actin binding protein	54443	2.71*E* + 22
12	*TPX2*	TPX2 microtubule nucleation factor	22974	2.71*E* + 22
14	*CDKN3*	Cyclin-dependent kinase inhibitor 3	1033	2.71*E* + 22
15	*CENPE*	Centromere protein E	1062	2.71*E* + 22
15	*RAD51AP1*	RAD51-associated protein 1	10635	2.71*E* + 22
17	*MCM2*	Minichromosome maintenance complex component 2	4171	2.71*E* + 22
18	*ATAD2*	ATPase family AAA domain-containing 2	29028	2.71*E* + 22
18	*FANCI*	FA complementation group I	55215	2.71*E* + 22
18	*SMC4*	Structural maintenance of chromosomes 4	10051	2.71*E* + 22

**Table 2:** Intergene correlation analysis of expression levels of *MCM2*, *NUSAP1*, *EGFR*, *ERBB2*, and *KRAS*.

GEPIA dataset			ICGC dataset			TCGA dataset		
Gene 1	Gene 2	Correlation coefficient	Gene 1	Gene 2	Correlation coefficient	Gene 1	Gene 2	Correlation coefficient
*MCM2*	*NUSAP1*	0.9	*MCM2*	*NUSAP1*	0.879	*MCM2*	*NUSAP1*	0.9
*EGFR*	*NUSAP1*	0.6	*MCM2*	*ERBB2*	0.704	*MCM2*	*ERBB2*	0.8
*ERBB2*	*NUSAP1*	0.8	*MCM2*	*KRAS*	0.78	*MCM2*	*KRAS*	0.8
*KRAS*	*NUSAP1*	0.82	*MCM2*	*EGFR*	0.75	*MCM2*	*EGFR*	0.62
*KRAS*	*MCM2*	0.8	*NUSAP1*	*ERBB2*	0.705	*NUSAP1*	*ERBB2*	0.8
*ERBB2*	*MCM2*	0.8	*NUSAP1*	*KRAS*	0.76	*NUSAP1*	*KRAS*	0.82
*EGFR*	*MCM2*	0.62	*NUSAP1*	*EGFR*	0.692	*NUSAP1*	*EGFR*	0.6
*KRAS*	*KRAS*	0.82	*KRAS*	*ERBB2*	0.748	*KRAS*	*ERBB2*	0.82
*EGFR*	*KRAS*	0.76	*KRAS*	*EGFR*	0.86	*KRAS*	*EGFR*	0.76
*EGFR*	*KRAS*	0.72	*ERBB2*	*EGFR*	0.673	*ERBB2*	*EGFR*	0.72

## Data Availability

The data used to support the findings of this study are available from the corresponding author upon request.

## Supplementary Materials

Supplementary 1 Fig. S1: data preprocessing. (a) Box plot of standardized expression data for the GSE15471 dataset. (b) Box plot of the standardized expression data for the GSE16515 dataset. The red symbol indicates a normal tissue sample, and the blue symbol indicates a sample from a patient with PC. The red diamond in each box represents the average level of gene expression in each sample. (c) Sample clustering map of the GSE15471 dataset. (d) Sample clustering map of the GSE16515 dataset. Blue represents downregulation of genes, and red represents upregulation of genes.

Supplementary 2 Fig. S2: GO and KEGG pathway enrichment analyses of the hub genes. (a) BP, (b) CC, (c) MF, and (d) KEGG analysis results.

Supplementary 3 Fig. S3: genetic variation analysis of the hub genes. (a) Changes in the hub gene copy number and a mutation panorama. (b) Mutation details of the hub genes.

Supplementary 4 Fig. S4: association of hub gene expression with disease-free survival (DFS) of patients with PC. (a) *BUB1*, (b) *CDK1*, (c) *FANCI*, (d) *KIF20A*, (e) *HMMR*, (f) *KIF23*, (g) *MCM2*, (h) *NUSAP1*, and (i) *TPX2*. The solid line represents the survival curve, and the dashed line represents the 95% confidence interval. Patients with higher than the median value are indicated by the red line, and those with lower than the median value are indicated by the blue line. Log-rank *P* < 0.05 was considered statistically significant.

Supplementary 5 Fig. S5: dot plots of MCM2 and NUSAP1 expression in different tumor and normal specimens. Each point represents a sample, with red representing tumor samples and green representing normal samples. (a) MCM2 expression. (b) NUSAP1 expression.

Supplementary 6 Fig. S6: mRNA and protein expression of MCM2 and NUSAP1 in normal human tissues, based on the Human Protein Atlas. (a) *MCM2* mRNA. (b) *NUSAP1* mRNA. (c) MCM2 protein. (d) NUSAP1 protein.
