# Supplementary figures and images for: MCM2 and NUSAP1 Are Potential Biomarkers for the Diagnosis and Prognosis of Pancreatic Cancer
Source: Biomed Res Int. 2020 Apr 28;2020:8604340. doi: 10.1155/2020/8604340 (PMC7206867; doi:10.1155/2020/8604340)

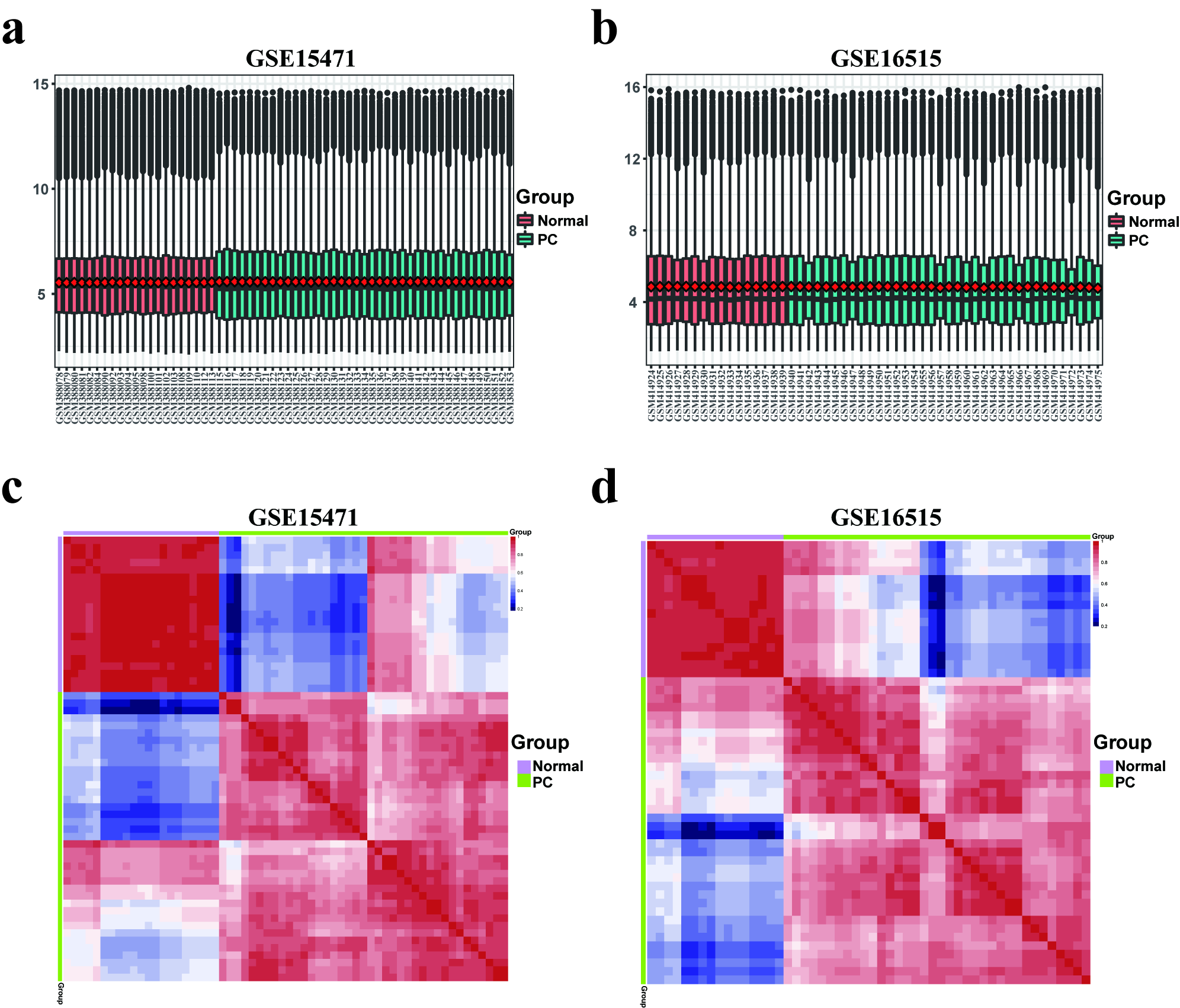

Supplement: Supplementary 1 — Fig. S1: data preprocessing. (a) Box plot of standardized expression data for the GSE15471 dataset. (b) Box plot of the standardized expression data for the GSE16515 dataset. The red symbol indicates a normal tissue sample, and the blue symbol indicates a sample from a patient with PC. The red diamond in each box represents the average level of gene expression in each sample. (c) Sample clustering map of the GSE15471 dataset. (d) Sample clustering map of the GSE16515 dataset. Blue represents downregulation of genes, and red represents upregulation of genes. [file 8604340.f1.tif]

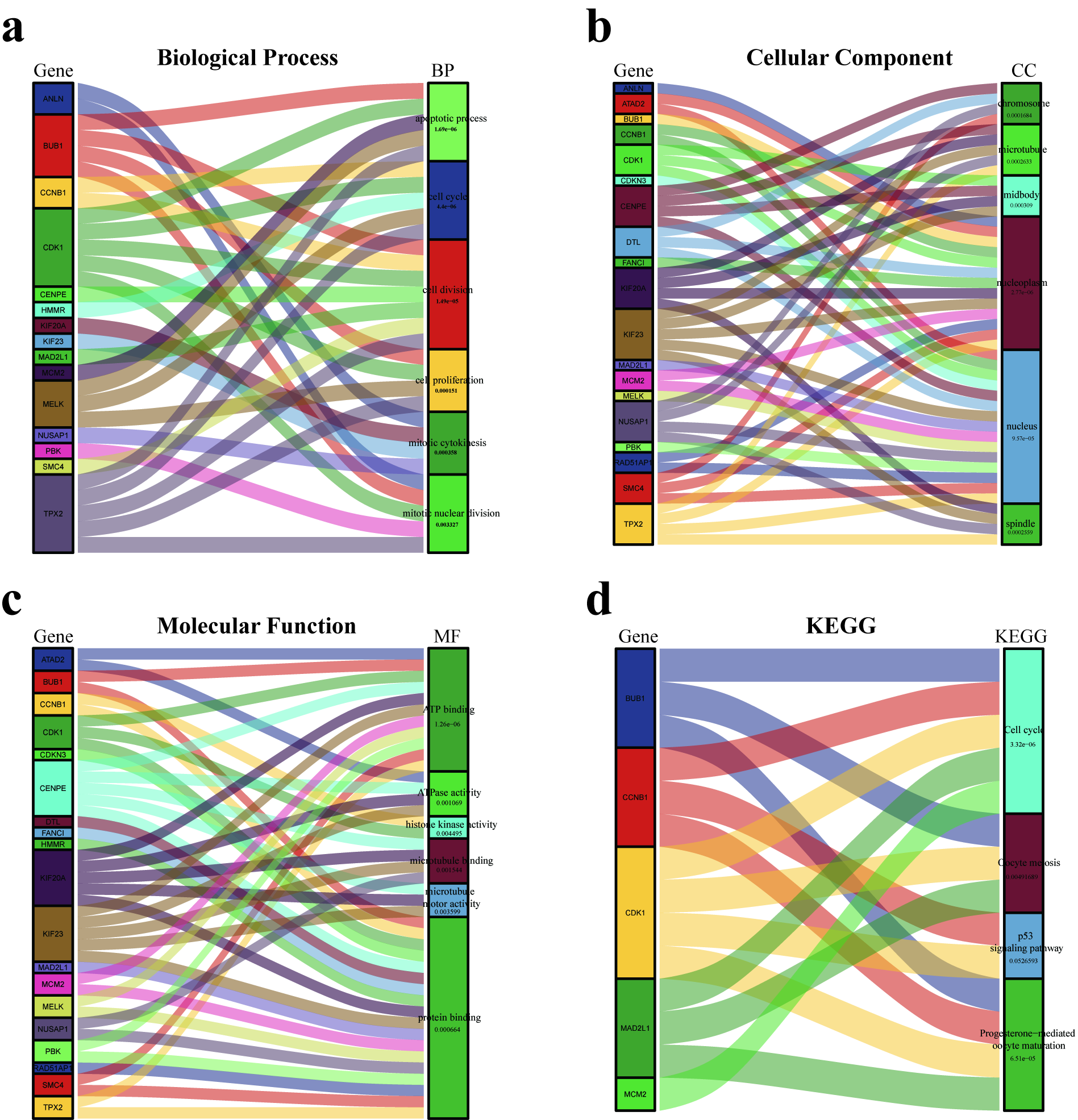

Supplement: Supplementary 2 — Fig. S2: GO and KEGG pathway enrichment analyses of the hub genes. (a) BP, (b) CC, (c) MF, and (d) KEGG analysis results. [file 8604340.f2.tif]

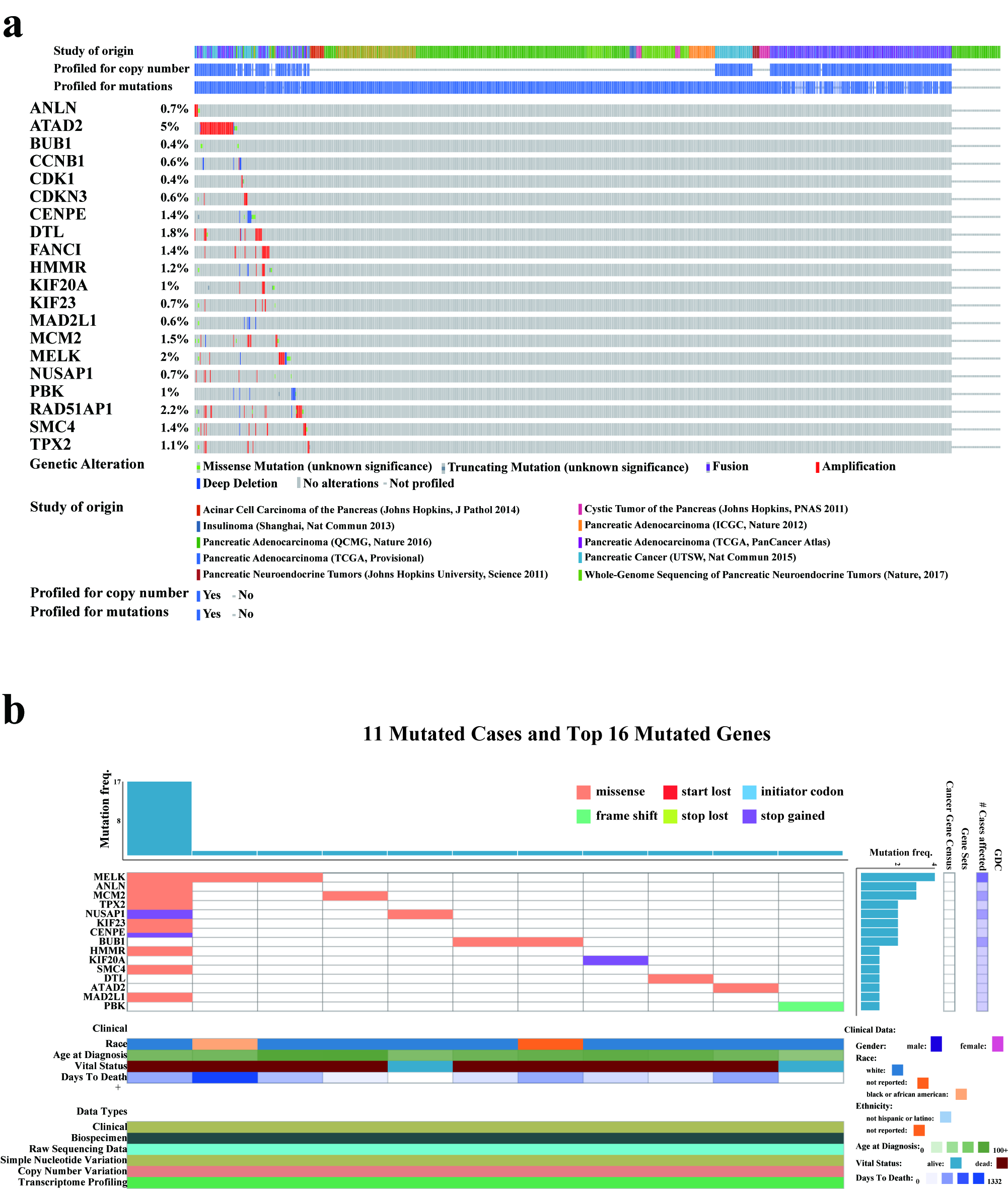

Supplement: Supplementary 3 — Fig. S3: genetic variation analysis of the hub genes. (a) Changes in the hub gene copy number and a mutation panorama. (b) Mutation details of the hub genes. [file 8604340.f3.tif]

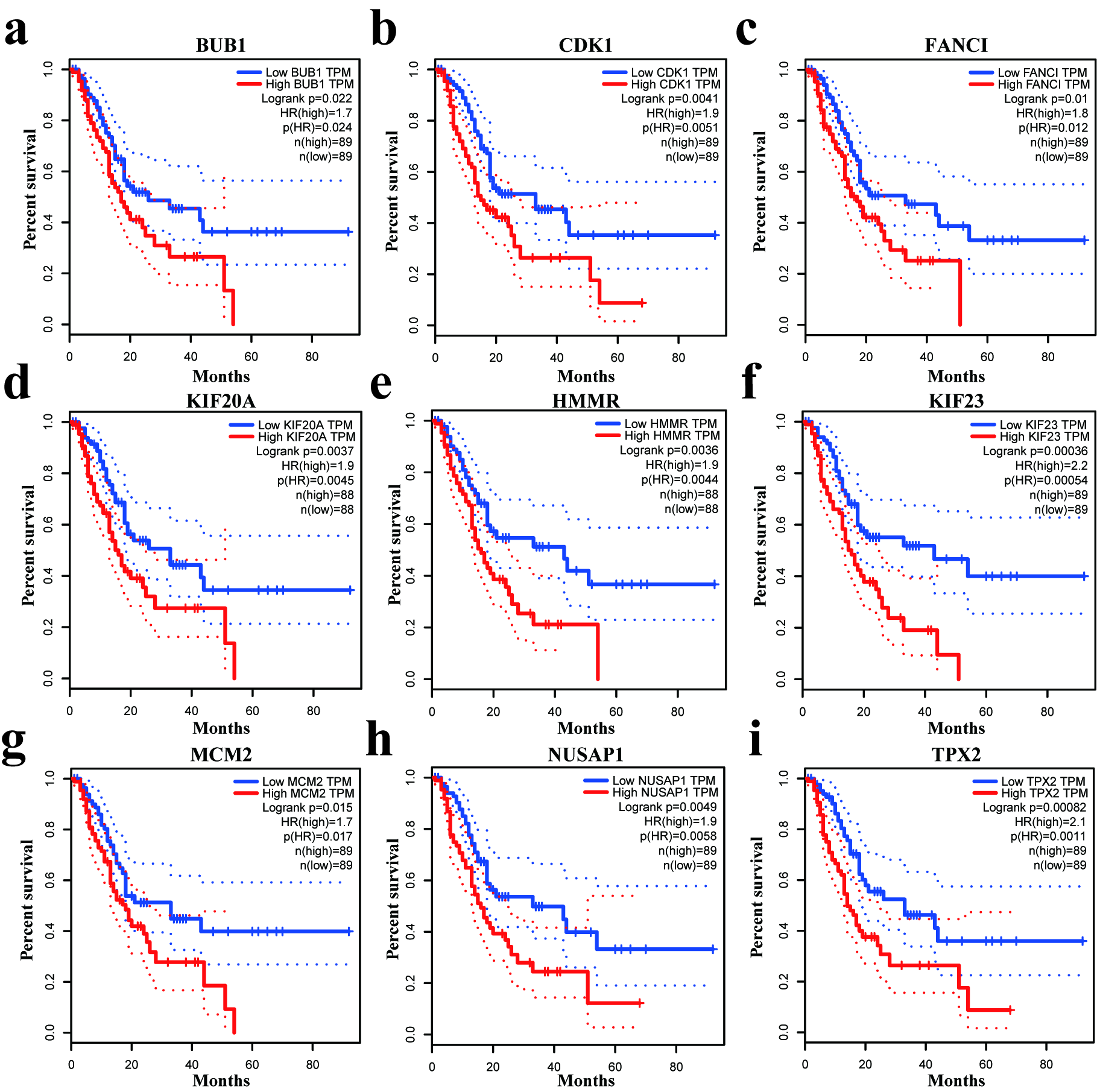

Supplement: Supplementary 4 — Fig. S4: association of hub gene expression with disease-free survival (DFS) of patients with PC. (a) BUB1, (b) CDK1, (c) FANCI, (d) KIF20A, (e) HMMR, (f) KIF23, (g) MCM2, (h) NUSAP1, and (i) TPX2. The solid line represents the survival curve, and the dashed line represents the 95% confidence interval. Patients with higher than the median value are indicated by the red line, and those with lower than the median value are indicated by the blue line. Log-rank P < 0.05 was considered statistically significant. [file 8604340.f4.tif]

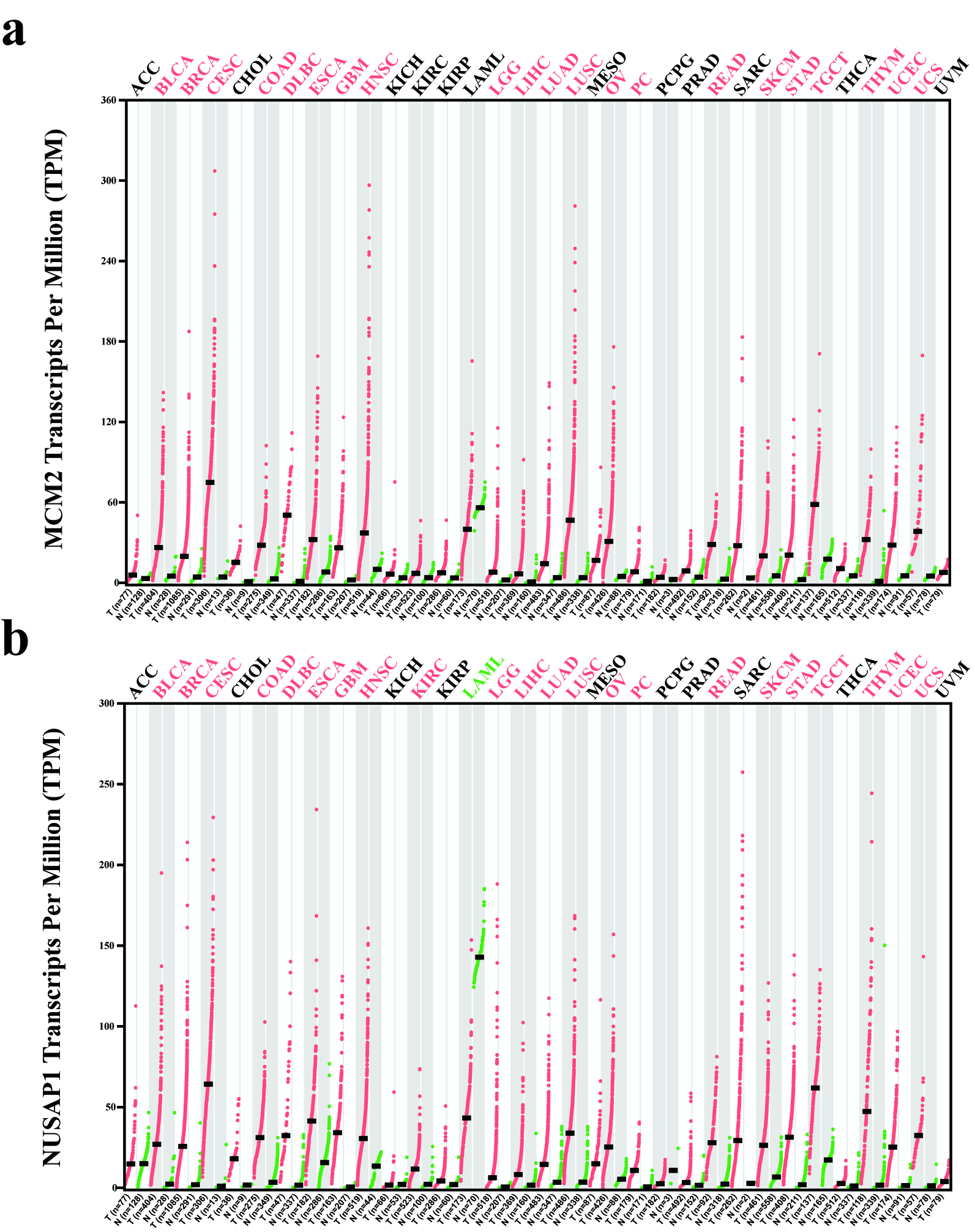

Supplement: Supplementary 5 — Fig. S5: dot plots of MCM2 and NUSAP1 expression in different tumor and normal specimens. Each point represents a sample, with red representing tumor samples and green representing normal samples. (a) MCM2 expression. (b) NUSAP1 expression. [file 8604340.f5.tif]

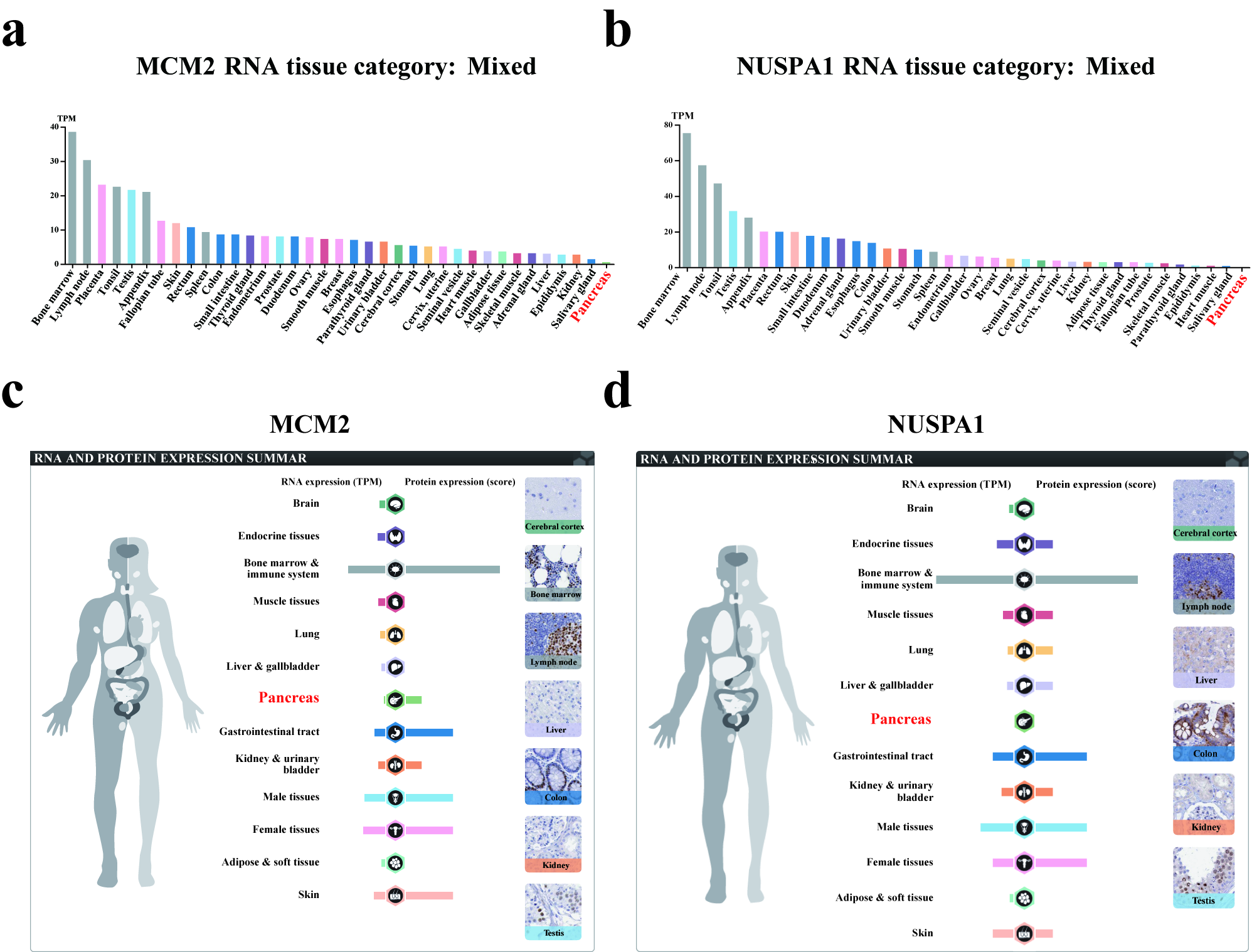

Supplement: Supplementary 6 — Fig. S6: mRNA and protein expression of MCM2 and NUSAP1 in normal human tissues, based on the Human Protein Atlas. (a) MCM2 mRNA. (b) NUSAP1 mRNA. (c) MCM2 protein. (d) NUSAP1 protein. [file 8604340.f6.tif]
